# The Cloning and Expression of Human Monoclonal Antibodies: Implications for Allergen Immunotherapy

**DOI:** 10.1007/s11882-015-0588-z

**Published:** 2016-01-16

**Authors:** Louisa K. James

**Affiliations:** Randall Division of Cell and Molecular Biophysics and MRC and Asthma UK Centre for Allergic Mechanisms of Asthma, King’s College London, SE1 1UL London, UK

**Keywords:** Human monoclonal antibodies, Allergen immunotherapy, B cells, Cloning, Human, Allergy

## Abstract

Allergic responses are dependent on the highly specific effector functions of IgE antibodies. Conversely, antibodies that block the activity of IgE can mediate tolerance to allergen. Technologies that harness the unparalleled specificity of antibody responses have revolutionized the way that we diagnose and treat human disease. This area of research continues to advance at a rapid pace and has had a significant impact on our understanding of allergic disease. This review will present an overview of humoral responses and provide an up-to-date summary of technologies used in the generation of human monoclonal antibodies. The impact that monoclonal antibodies have on allergic disease will be discussed, with a particular focus on allergen immunotherapy, which remains the only form of treatment that can modulate the underlying immune mechanisms and induce long-term clinical tolerance.

## Introduction

Allergen immunotherapy (AIT) was introduced over a century ago with the first clinical trial conducted by Noon and Freeman in 1911 [1, 2]. The aim of AIT is the induction of clinical tolerance to a sensitizing allergen and has proven highly effective in the treatment of rhinitis, asthma, and venom allergy. Conventional AIT involves the subcutaneous injection of increasing doses of allergen extract over many years. Treatment carries a risk of inducing adverse side effects including systemic anaphylaxis [3]; hence, a major goal of research into AIT has been to improve both safety and efficacy. To this end, alternative routes of allergen administration have or are being successfully adopted (e.g., sublingual, oral, epicutaneous, intralymphatic). Novel vaccine design has led to the development of alternative forms of treatment including peptide immunotherapy, immunotherapy with recombinant allergens or modified allergen extracts, and the use of adjuvants that stimulate innate immune receptors. In addition, the combined use of monoclonal antibody therapy alongside AIT has proven highly successful. Monoclonal antibodies (mAbs) have revolutionized the way we diagnose and treat human disease. Of the mAbs now approved or under review, the overwhelming majority are licensed for use in cancer or autoimmune diseases. Until recently, the only mAb in clinical use for allergic disease was the anti-IgE antibody Omalizumab (Xolair), which was approved in the USA in 2003 and in Europe in 2005 for patients with asthma. In addition, the anti-IL-5 antibody Mepolizumab has now been approved for use in severe eosinophilic asthma. Rapid technological advances in medicine over recent years provide an opportunity to reassess our understanding of allergic diseases and our approach to AIT. The aims of this review are to assess how advances in monoclonal antibody technology could impact the field of allergy and in particular address some of the challenges of allergen immunotherapy.

## The Breadth of the Humoral Response

**Fig. 1:**
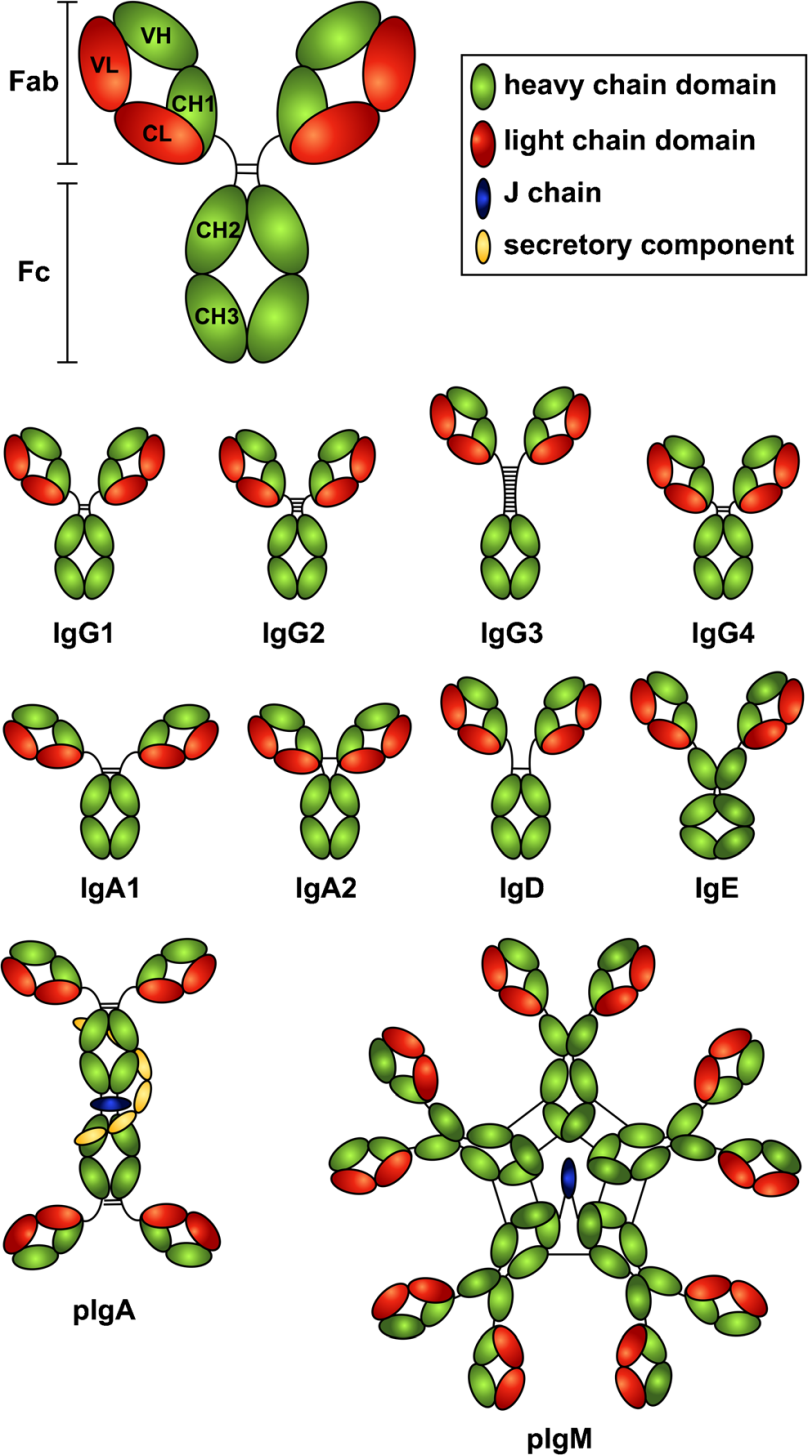
Human Immunoglobulins. Schematic representation of the human immunoglobulin subclasses, monomeric IgG1, IgG2, IgG3, IgG4, IgA1, IgA2, IgD, IgE, and polymeric (*p*) IgA and IgM

Antibodies are secreted glyco-proteins that recognize and bind to antigen with exquisite specificity through the highly variable fragment antigen binding (Fab) region (Fig. 1). The various effector functions of antibodies are elicited via the fragment crystallizable (Fc) region (Fig. 1), through engagement with Fc receptors and other components of the immune system.

Human antibodies are encoded within the heavy chain immunoglobulin locus on chromosome 14 (V_H_, D, J_H_, and C_H_), the kappa (κ) light chain immunoglobulin locus on chromosome 2 (Vκ, Jκ and Cκ), and the lambda (λ) light chain locus on chromosome 22 (Vλ, Jλ, and Cλ). Antibodies are produced by B cells which develop in the bone marrow and express a membrane version of the antibody in the form of a B cell receptor or membrane immunoglobulin. Following activation by antigen in the periphery, specific B cells undergo a series of processes that involve clonal proliferation, isotype switching (or class switch recombination), and affinity maturation, whereby somatic hypermutation of variable region genes leads to changes in the affinity of the BCR. B cells that bind to antigen with high affinity receive survival signals and may differentiate into memory B cells or plasma cells, the latter of which gives rise to secretion of high affinity antibodies. Long-lived, terminally differentiated plasma cells migrate to the bone marrow where they are thought to produce in excess of 10^8^ antibody molecules per hour [4]. Human antibodies are grouped into five classes (IgD, IgM, IgA, IgE, and IgG, Fig. 1) with IgA and IgG further divided into two and four subclasses (IgA1 and IgA2, IgG1, IgG2, IgG3, and IgG4), respectively, [5].

IgM is expressed as a monomer on the cell surface during B cell development. Following maturation, IgM is secreted by activated B cells as a pentamer or, less commonly as a hexamer, with the addition of the J-chain (Fig. 1). The multimeric structure of IgM increases avidity of antigen binding thus enhancing its functional activities including opsonization and complement fixation. IgM comprises around 10 % of serum antibodies and is important for primary immune responses [6]. A significant proportion of IgM is of low affinity and often polyreactive; this latter property may be important for broad recognition of pathogens during primary responses. IgD is co-expressed with IgM on naïve B cells prior to isotype switching. Although IgD^+^ B cells can differentiate into plasma cells, this monomeric antibody comprises less than 0.5 % of serum. The immune function of secreted IgD is poorly defined; however, class-switched IgM^−^IgD^+^ B cells resident in human respiratory mucosa are thought to play an important role in immunity to pathogens through direct interactions with basophils [7, 8]. IgG1 is the most well-studied and hence well-defined of the antibody subclasses owing to its abundance in serum, comprising around half of all serum antibodies. Like all IgG antibodies [9•], IgG1 is monomeric and is usually generated during secondary immune responses to protein antigens, mediating robust effector functions. IgG1 binds with high affinity to IgG receptors and efficiently triggers the complement cascade. IgG2, around 16 % of serum antibodies, dominates the antibody response to carbohydrates such that nearly all antibodies specific for bacterial polysaccharides are IgG2 [10]. IgG3 is the most potent IgG subclass in terms of effector function, due to its exceptional ability to activate complement and bind to IgG receptors. Like IgG1, it is generated in response to protein antigens but is present at much lower concentrations, comprising just 5 % of serum antibodies. IgG3 has an unusually long hinge consisting of 62 amino acids compared to IgG2, IgG4 (both 12 amino acids), and IgG1 (15 amino acids). IgG4 is the least abundant IgG subclass comprising just 3 % of serum antibodies. It appears following long-term exposure to antigen and is generally regarded as an anti-inflammatory antibody, often associated with immune tolerance [11]. In addition, IgG4 is unable to fix complement and binds with lower affinity to IgG receptors than IgG1 [12•]. Conversely, the spectrum of conditions known as IgG4-related disease is characterized by high concentrations of serum IgG4 and inflammatory infiltration of IgG4^+^ plasma cells into tissue [13]. IgA1 and IgA2 are produced predominantly at mucosal surfaces where they are secreted in a dimeric form in combination with a J-chain and an additional polypeptide, the secretory component (Fig. 1) that protects the antibodies from proteolytic degradation. In serum, IgA exists as a monomer with IgA1 accounting for around 13 % of serum antibodies and IgA2 3 %. IgA2 is the predominant subclass in secretions and gut-associated lymphoid tissue whereas IgA1 is produced at higher proportions in secondary lymphoid tissue [14]. Secretory IgA1 and IgA2 are poor at activating inflammatory immune responses and are unable to fix complement but rather act to neutralize antigens through the process of immune exclusion. In contrast, serum IgA1 can initiate effective immune responses following binding to FcαRI on the surface of granulocytes. IgE is the least abundant isotype in human serum comprising less than 0.01 % of serum antibody in non-atopic individuals and may be up to ten times higher in allergic individuals, though still low relative to other isotypes. This low abundance is attributed to the potent pro-inflammatory immune responses elicited by IgE through interaction with its receptors FcεRI and FcεRII (CD23) expressed on the surface of granulocytes and antigen-presenting cells. IgE is well-known for its central role in allergic disease [15].

Since their first description by Paul Ehrlich over a century ago, our understanding of human antibodies has revealed the incredible diversity that these molecules possess. Human antibodies have broad effector functions that can act at every site of the human body, often with precise specificity for antigen targeting. As the basis of humoral immunity, antibodies provide protection against infectious disease, regulate immune responses, and maintain immune homeostasis. It is perhaps no surprise that we have realized the potential of antibodies for the benefits of human health.

## The Advent of Therapeutic Monoclonal Antibodies for Human Disease

In 1984, Georges Köhler and Cesar Milstein were awarded the Nobel Prize for Medicine for their development of hybridoma technology nearly a decade previously [16]. This pioneering technique provided the tools for the generation of monoclonal antibodies, mAbs (i.e., antibodies of a single specificity) and ultimately for their diverse application in scientific research and in diagnostic and therapeutic medicine. Hybridoma technology involves the fusion of B cells from immunized mice with myeloma cells to produce an immortalized cell line capable of continuous production of mAbs specific for a desired target. Early clinical studies with hybridoma-derived mAbs found that therapeutic administration resulted in the development of immune responses directed towards the murine proteins. These responses caused either a failure of therapy due to the blocking or neutralizing effects of the anti-mAb, anti-idiotypic response [17] or immediate or delayed hypersensitivity reactions. Efforts to overcome these adverse effects were developed during the 1980s following the advent of recombinant technology. One such approach involved the production of chimeric antibodies in which the murine constant regions were replaced with human constant regions using molecular cloning [18, 19]. An added advantage of the incorporation of a human Fc was their ability to interact with human Fc receptors and complement components thereby facilitating cooperation with the immune system. Although this represented a significant improvement in terms of treatment efficacy, chimeric antibodies also elicit anti-idiotypic responses. Subsequently, humanized antibodies were developed in which the hypervariable loops (CDR) from murine antibodies were grafted onto human framework (FR) regions [20] resulting in a marked reduction in immunogenicity. The first mAb licensed for use in humans was muromonab, a murine IgG2a antibody generated by hybridoma technology, which targets human CD3 to treat severe steroid-resistant transplant rejection [21]. Although effective, muromonab caused significant adverse effects due to (i) stimulation of potent T cell responses, (ii) the production of anti-mouse antibodies, reducing the efficacy of the treatment, and (iii) isolated cases of anaphylaxis. These ultimately led to its withdrawal from the market. Since then, 45 mAbs have been approved for use in humans in the USA. A further four are currently under review and many more in clinical trials. The overwhelming majority of these are chimeric or humanized forms. Despite the success yielded by humanization of mAbs, this technically challenging approach often resulted in reduced affinity binding of the antibody to its target, the development of fully human antibodies was therefore considered an attractive alternative approach.

## The Development of Human Monoclonal Antibodies

In 1985, following the development of transgenic mice, Alt and colleagues suggested that such technology could eventually be used to “engineer a mouse to make specific human antibodies,” they wrote that “although conceptually outlandish, such genetic programming experiments may actually be realized in the not too distant future.” [22] Their predictions were correct and less than a decade later the first publications described the generation of fully human mAbs from transgenic mice [23, 24]. This approach involves replacing the entire antibody repertoire of the mouse with genes encoding human antibodies. Following immunization, standard hybridoma techniques can be employed to generate fully human monoclonal antibodies. Like traditional hybridomas, this technique is limited to immunogenic, non-toxic antigens but takes advantage of the in vivo affinity maturation process, resulting in highly specific antibodies.

Alongside the development of transgenic technology, phage display provided a novel complementary approach for the development of human monoclonal antibodies. Phage display was first described in 1985 [25] and later used to generate libraries encoding antibody genes [26]. Antibody repertoires from humans or immunized animals are generated by PCR amplification of the variable regions of heavy and light chain fragments. The PCR products are then cloned into a phage display vector, and DNA encoding a single antibody fragment is ligated into the pIII (minor) or PVIII (major) coat protein gene of a single filamentous bacteriophage (such as M13). The phage is then transduced in *E. coli*, allowing expression of the antibody fragment as a fusion protein attached to the capsid. In this way, up to 10^8^ different antibody fragments may be displayed in a phage “library.” Antigen-specific antibody fragments are identified through the process of panning whereby target antigens are immobilized, allowing binding and enrichment of specific antibody fragments [27]. Importantly, the sequence of the variable regions can be determined thus providing a link between antibody genotype (i.e., sequence) and phenotype (i.e., specificity or affinity).

## Human B Cells as a Source of Monoclonal Antibodies

Immortalization with Epstein-Barr virus was one of the first techniques to allow the generation of monoclonal antibodies directly from human B cells [28]. Although this method proved useful in a research setting, the low efficiency of transformation limited its use for antibody discovery. An improved method for EBV transformation was developed in the laboratory of Antonio Lanzavecchia, in which the addition of TLR9 agonists (such as CpG) led to more efficient transformation of memory B cells [29].

Alternative approaches have since been developed to isolate memory B cells from peripheral blood and then isolate and clone the antibody variable regions using single-cell expression cloning [30]. By using labeled antigens, specific memory B cells can be directly isolated by flow cytometry to generate human monoclonal antibodies with desired specificities [31]. This method of single B cell cloning has resulted in the identification of numerous monoclonal antibodies specific for diverse targets including viruses [32, 33], parasites [34], and allergens [35••, 36]. Adaptations to this method include high-throughput culture of single B cells isolated from blood [37], which allows pre-screening of secreted antibodies in culture supernatant, so that only cells with desired specificities are subject to cloning and recombinant expression.

The development of high-throughput sequencing technology provided a novel approach to mine antibody repertoires with unprecedented depth [38]. This involves amplification of the antibody variable region genes followed by high-throughput sequencing. Typically, peripheral blood is used as a source of B cells for sequencing but has also been applied to determine immunoglobulin repertoires in mucosal tissues [39]. Immunoglobulin repertoire analysis is useful for analyzing the ancestral relationships between related clones and for examining the diversity and selection of antibodies. Although this approach has potential for identifying dominant clones of a given isotype, the ability to sequence only the heavy or light chains independently meant that information on the natural pairing of the respective chains is lost. Recent approaches have been developed to overcome this limitation. Reddy et.al [40] took advantage of the expansion of bone marrow plasma cells following immunization or infection to identify and express the most abundant V_H_ and V_L_ genes within the immunoglobulin repertoire. This resulted in a higher efficiency of antigen-specific antibodies than could be obtained through random pairing. The use of PCR to physically link IgH and IgL sequences from individual cells provides another method of retaining the natural pairing of heavy and light chains [41••]. In this approach, messenger RNA (mRNA) molecules are captured in an emulsion inside which the entire V_H_ and V_L_ regions are amplified by PCR then pooled for sequencing. A similar approach using “cell-barcoding” has also been employed for paired V_H_:V_L_ repertoire analysis; here, unique molecular barcodes are incorporated by “template-switching” during reverse transcription of mRNA from single B cells. This novel method was used to clone and express monoclonal auto-antibodies from the peripheral blood of patients with rheumatoid arthritis [42••] and *Staphylococcus aureus* antibodies isolated from patients with *S. aureus* bacteremia [43•].

## Novel Approaches to Allergen Immunotherapy

For over a century, allergen immunotherapy (AIT) has remained the only form of treatment that can modulate the underlying mechanisms of allergic disease. Currently, AIT is used to treat allergic rhinitis, allergic conjunctivitis, allergic asthma, and insect venom allergy. Both subcutaneous (SCIT) and sublingual (SLIT) forms of treatment are approved, though alternative routes (e.g., intralymphatic [44] and epicutaneous [45]) are under evaluation. In particular, over the last decade, the potential benefit of oral immunotherapy (OIT) for the treatment of food allergy has been assessed in a large number of clinical trials [46]. Although the efficacy of AIT is well-documented, it is regarded as an “underused” form of treatment for allergic diseases [47] in part due to the lack of standardized treatment regimens which results in variations in allergen preparations and differences in clinical practices from region to region. Several other novel approaches to improve the efficacy and safety of AIT have been evaluated, such as the use of recombinant allergens, including engineered modified “hypoallergens,” the use of novel adjuvants to target allergenic proteins more precisely to immune modulatory pathways or co-treatment with other anti-allergic drugs [48].

The use of conventional SCIT alongside anti-IgE treatment has been assessed in a number of clinical trials. Omalizumab is a humanized monoclonal antibody directed against the Cε3 domain of the constant (Fc) region of IgE (trade name Xolair, Novartis) [49]. It is administered intra-venously or subcutaneously and binds specifically to human IgE preventing interactions with the high-affinity receptor FcεRI. Due to steric hindrance, omalizumab cannot bind to receptor-bound IgE and thus does not induce cross-linking of effector cells. Omalizumab has proven efficacy in moderate and severe allergic asthma, and in seasonal and perennial allergic rhinitis [50]. A single dose results in a decrease of serum IgE and consequently a downregulation of FcεRI expression on mast cells and basophils [51]. The use of anti-IgE treatment alongside AIT was proposed on the basis that it would reduce the significant risk of systemic anaphylaxis that is associated with AIT. Indeed, pre-treatment with anti-IgE has been shown to significantly reduce the incidence of adverse reactions, particularly during the updosing phase of treatment and results in a greater reduction in symptoms than AIT alone [52–56]. While large clinical trials of anti-IgE and AIT have thus far been restricted to studies of allergic asthma and allergic rhinitis, recent smaller studies combining anti-IgE with OIT provide promise for this approach in the treatment of IgE-mediated food allergies [57, 58••]. Omalizumab is unsuitable for a significant proportion of patients who have high levels of serum IgE. Recently, however, a novel human anti-IgE mAb was generated that may be suitable for such patients. MEDI4212 was produced by phage display and affinity matured using combinatorial mutagenesis of the CDR regions, generating an antibody with 100-fold increased affinity for IgE compared to omalizumab [59•].

The combination of monoclonal antibody therapy with AIT has been explored with other targets of the allergic response. IL-4 is a prototypic T helper 2 (Th2) cytokine produced primarily by CD4^+^ T cells as well as by basophils. The importance of IL-4 in Th2-mediated inflammation and the induction of IgE class switching prompted the generation of monoclonal antibodies designed to neutralize IL-4. Like other mAbs-targeting single cytokines (e.g., anti-IL-5 and anti-IL-13), the results of clinical trials were disappointing, with variable levels of efficacy as a standalone treatment [60]. More recently, the use of anti-IL-4 in combination with AIT for allergic rhinitis was assessed in a double-blind study of 37 patients with seasonal allergic rhinitis [61]. Despite an apparent reduction in the numbers of IL-4-producing cells in peripheral blood, the concomitant use of anti-IL-4 with AIT had no additional beneficial effects on the clinical response. The lack of efficacy of anti-IL-4, in contrast to the synergistic effects of anti-IgE in combination with AIT, may reflect the important role of the humoral response in both allergy and tolerance induction. The induction of blocking antibodies, particularly of the IgG4 subclass is an important mechanism in the efficacy of AIT. In fact, blocking antibody responses have proven therapeutic properties in patients with severe allergic disease.

## The Induction of Blocking Antibodies by AIT

In 1937, Robert Cooke and Mary Loveless demonstrated that co-administration of serum from subjects who had received multiple subcutaneous injections of ragweed pollen over 3–12 months, could inhibit immediate allergic responses in the skin, elicited by intradermal injection of ragweed with allergic serum [62]. These early experiments demonstrated that “blocking antibodies” are able to inhibit the activity of IgE in an allergen-specific manner. In 1978, Maurice Lessof and colleagues took this concept a step further when they demonstrated that an infusion of gamma-globulin from “hyperimmune” beekeepers, with high levels of anti-phospholipase IgG protected against systemic anaphylaxis. Administration of “protective serum” to five subjects with bee venom allergy lowered the threshold dose required to induce systemic reactions by 1.5 to 5 times the amount that was previously required. [63].

Later, similar studies [64, 65] provide proof of concept that administration of allergen-specific blocking antibodies may represent a valid therapeutic approach for allergic disease, one that is more recently gaining favor following the advent of monoclonal antibody technology [66]. An important consideration for this approach is the polyclonal nature of IgE responses, which involve multiple epitopes often on several different allergenic proteins. Thus, a monoclonal antibody directed against a single allergenic epitope would be unlikely to provide a similar level of passive protection that can be achieved with hyperimmune serum. The use of multiple combinations of monoclonal antibodies is being evaluated for several human diseases [67]. In a murine model of allergic asthma, administration of a “polyclonal” mixture of seven recombinant ovalbumin (OVA)-specific IgG2b antibodies to OVA-sensitized mice provided a better level of protection against OVA-induced airway inflammation and hyper-reactivity than a single monoclonal antibody [68].

## Role for Allergen-Specific Antibodies in Vaccine Design

The availability of well-characterized allergen-specific antibodies could have broad applications for our understanding and treatment of allergic diseases, from direct therapeutic application as passive immunotherapy to the standardization of allergen extracts for AIT. In addition, allergen-specific monoclonal antibodies can facilitate the design of novel forms of allergen immunotherapy. Inhibiting the activity of IgE has clear benefits in terms of improving the safety and efficacy of AIT, exemplified by the concomitant use of anti-IgE. It follows that hypoallergens (i.e., allergens which lack IgE epitopes) would make excellent candidates for allergen immunotherapy. Early attempts to alter the allergenicity of immunotherapy vaccines through chemical modification (allergoids) had limited success due to lack of immunogenicity or residual IgE activity, but re-evaluation of this approach has led to a number of recent initiatives, most notably the EU-funded FAST project [69••, 70].

Understanding the molecular determinants of IgE recognition would allow the rational design of recombinant hypoallergens for AIT. Epitopes formed by discreet continuous regions of polypeptide (“linear” epitopes) can be deduced by epitope arrays [71]. However, the majority of epitopes from both inhaled and food allergens are discontinuous or “conformational,” formed by close association of amino acids in the three-dimensional structure. Such epitopes can only be defined by biophysical approaches such as X-ray crystallography or nuclear magnetic resonance (NMR). Of the known 827 allergens in the official WHO/IUIS database (www.allergen.org), protein database (PDB) structures are available for 92 (11 %) of these [72]. In turn, the structures of 11 allergen-antibody complexes for six allergens (birch pollen, bee venom, cockroach, cow’s milk, house dust mite, and grass pollen) have been solved [73–79] (Table 1). All of these complexes are formed of discontinuous epitopes, except for hyaluronidase from bee venom (Api m 2) which comprises a continuous stretch of nine amino acids. However, the protruding loop structure formed by this region in the three-dimensional structure means that isolated peptides with an identical amino acid sequence are not recognized by the corresponding antibody or even by serum IgE [76]. Proof of principle for structure-based vaccine design is highlighted by studies on Bet v 1, the major allergen from birch pollen. Based on X-ray crystallographic analysis of the murine Bet v 1-specific mAb BV16 in complex with Bet v 1, Spangfort et.al*.* demonstrated that a single point mutation in the epitope of Bet v 1, abolishes binding of BV16 and reduces binding of serum IgE by 50 % [80]. Thus, point mutations in IgE epitopes, guided by analysis of antibody allergen complex structures, can direct the design of hypoallergenic proteins for therapy of IgE-mediated allergy.

**Table 1:** Allergen-antibody complex structures

Allergen	Source	mAb clone	Species	Isotype	Technology
Gal d 4	Hen’s egg	HyHEL-10	Mouse	IgG1	Hybridoma
Bet v 1	Birch pollen	BV16	Mouse	IgG1	Hybridoma
Bos d 5	Cow’s milk	D1	Human	IgE	Phage display
Api m 2	Bee venom	21E11	Mouse	IgG1	Hybridoma
Bla g 2	Cockroach	7C11	Mouse	IgG1	Hybridoma
Phl p 2	Grass pollen	HuMab2	Human	IgE	Phage display
Der f 1/Der p 1	House dust mite	4C1	Mouse	IgG1	Hybridoma

While the advent of component resolved diagnostics has revolutionized our understanding of patterns of sensitization, it has also highlighted the surprising disconnect between sensitization and reactivity and the considerable variation in IgE responses between individuals. The availability of allergen-specific antibodies directed against clinically relevant, allergenic epitopes would allow us to better understand the molecular determinants of allergenicity. Identification of dominant conformational epitopes through structural studies of antibody allergen complexes will ultimately lead to the engineering of recombinant hypoallergens for AIT.

## Conclusions

The list of antibodies approved or in clinical trials is constantly evolving as this form of treatment is adopted in broad areas of medicine. With rapid improvements in antibody engineering, antibody expression, and greater understanding of antibody effector functions, we have entered an era of rapid progress. Antibodies can now be made smaller (e.g., diabodies [81]), more stable (e.g., through hinge engineering [82]), more specific (e.g., in vitro affinity maturation [83]) and ultimately more efficacious. Our understanding of antibody responses in disease has improved as a direct result of high-throughput methods for isolation and characterization of human antibodies and computational approaches to understand how antibody repertoires are shaped. Given the central role of antibody responses in allergic disease, we may now take advantage of these advances to improve our understanding of antibody allergen interactions and identify disease-relevant epitopes of clinically relevant allergens. The success of anti-IgE will hopefully encourage further work into the potential role of monoclonal antibodies for allergic disease. This form of treatment has the potential to speed up the progressively advancing progress that has thus far been made in improving the safety and efficacy of AIT.
